# Supplementary figures and images for: Built environment and physical activity in adolescents: Use of the kernel density estimation and the walkability index
Source: PLoS One. 2024 Mar 19;19(3):e0299628. doi: 10.1371/journal.pone.0299628 (PMC10950253; doi:10.1371/journal.pone.0299628)

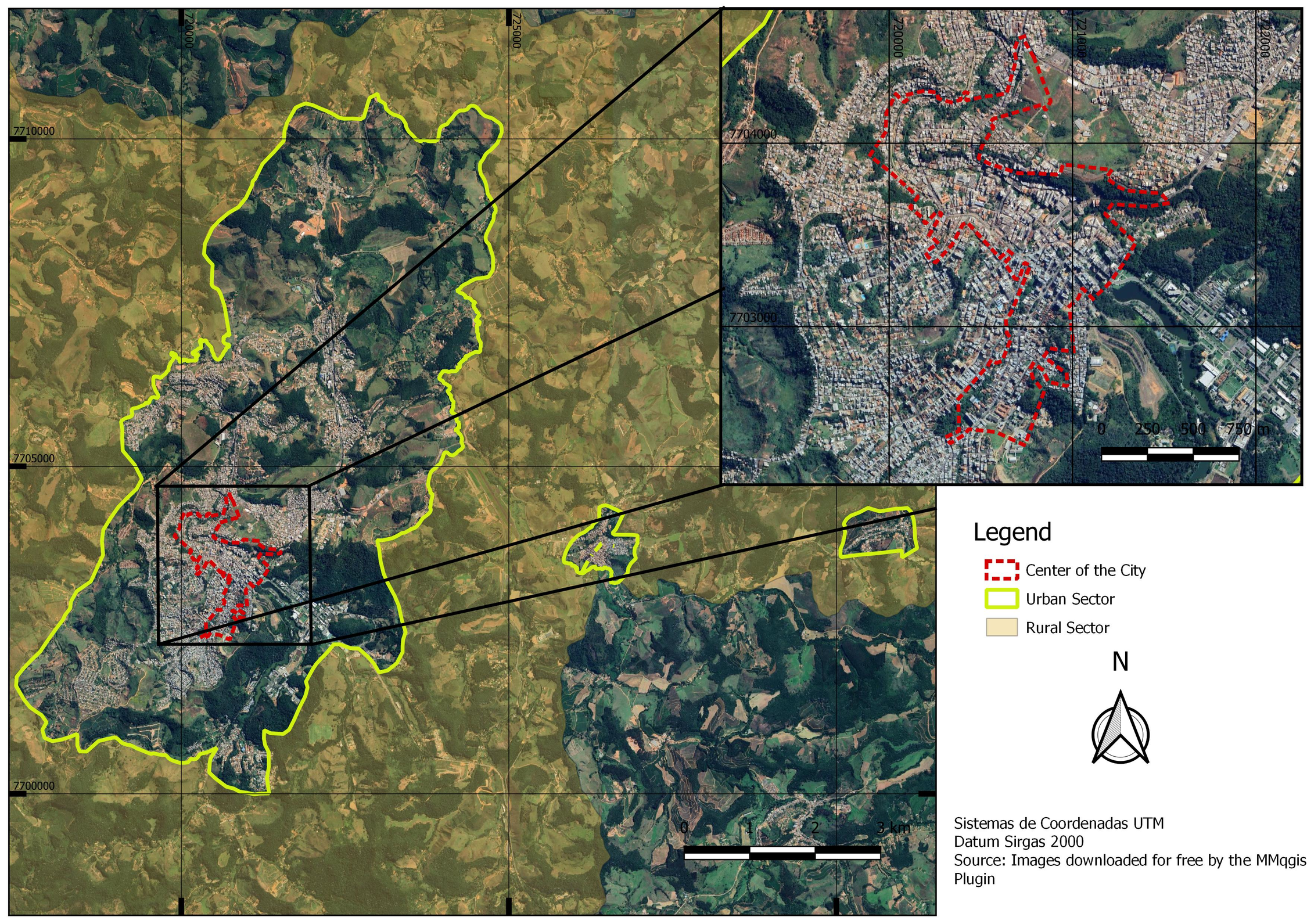

Supplement: S1 Fig — UTM; Universal Transverse Mercator System. (TIF) [file pone.0299628.s001.tif]

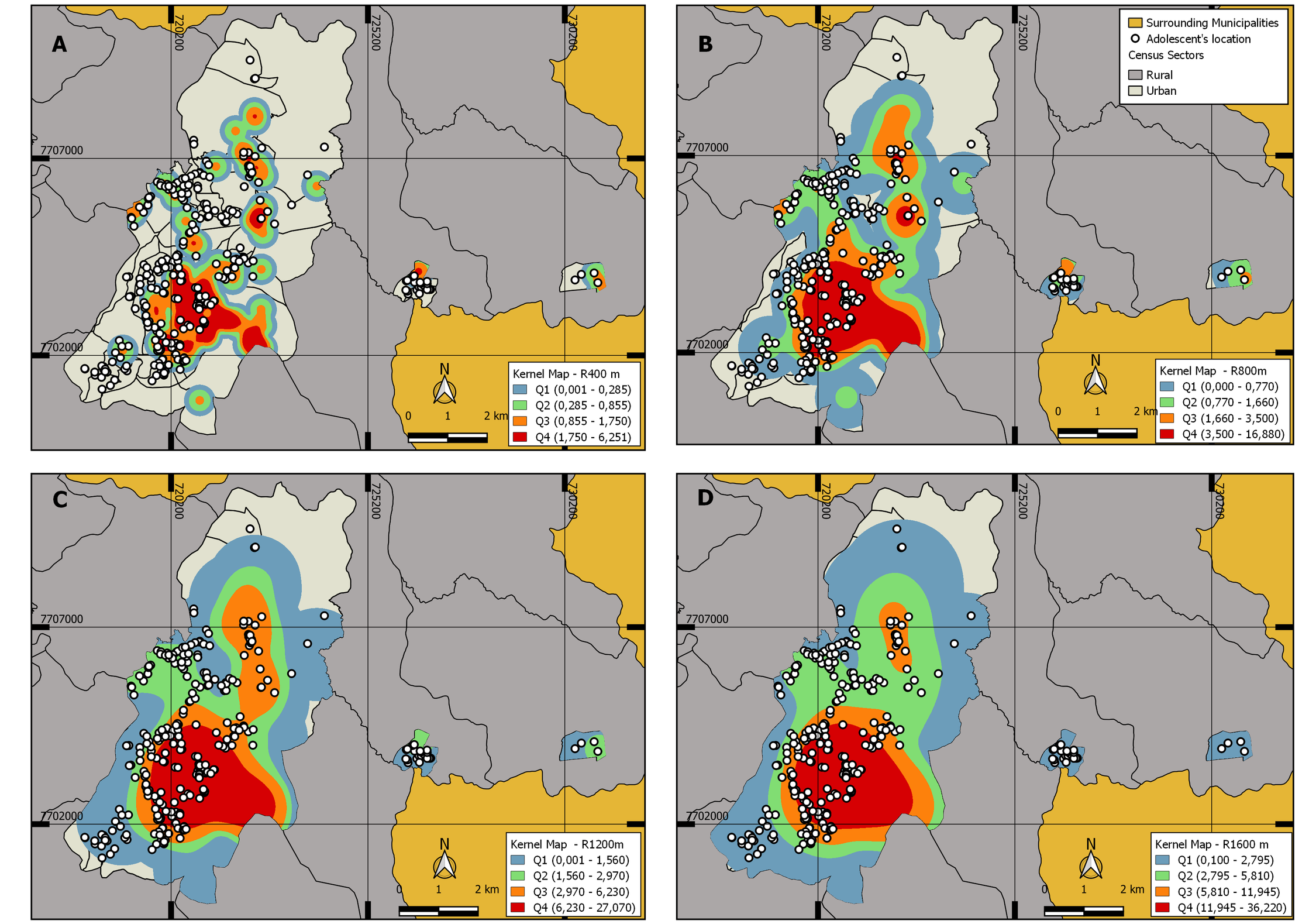

Supplement: S2 Fig — Fig 2A. Kernel Map—R400m, Kernel Map in Radius of 400 meters. Fig 2B. Kernel Map—R800m, Kernel Map in Radius of 800 meters. Fig 2C. Kernel Map—R1200m, Kernel Map in Radius of 1200 meters. Fig 2D. Kernel Map—R1600m, Kernel Map in Radius of 1600 meters. Q1, quartile 1; Q2, quartile 2; Q3, quartile 3; Q4, quartile 4; km, kilometer. Coloring of Quartiles in the Kernel Map: Q1, blue; Q2, green; Q3, orange; Q4, red. (TIF) [file pone.0299628.s002.tif]
