# Supplementary material for: Built environment and physical activity in adolescents: Use of the kernel density estimation and the walkability index
Source: PLoS One. 2024 Mar 19;19(3):e0299628. doi: 10.1371/journal.pone.0299628 (PMC10950253; doi:10.1371/journal.pone.0299628)
Supplement: S1 Table — un/km, unit per kilometer; %, percentage; SD, standard deviation; IQ, interquartile interval. (DOCX) [file pone.0299628.s003.docx]

**Supplementary Table 1. Descriptive measures of the variables related to the walkability index.**

| **Variables** | **Unit of measurement** | **Average ± SD** | **Minimum Value** | **Maximum Value** | **Average (IQ)** |
| --- | --- | --- | --- | --- | --- |
| *Residential Density* | un/km | 147.0 (±107.79) | 4.0 | 626.0 | 131.0 (70.5 – 193.0) |
| *Commercial Density* | un/km | 12.0 (±14.84) | 0.4 | 68.9 | 5,8 (2.7 – 13.3) |
| *Presence of Sidewalks* | % | 49.1 (±26.48) | 0 | 100 | 50 (31.1 – 70.9) |
| *Presence of public illumination* | % | 77.5 (±17.04) | 12 | 100 | 85 (69 - 89) |
| *Density of intersection of the streets* | un/km | 6.4 (±2.08) | 1.56 | 12.7 | 6.11 (5.2 – 7.6) |
| *Walkability index* | dimensionless | -0.02 (±2.95) | -10.84 | 6.93 | 0.13 (-1.80 – 1.54) |

un/km, unit per kilometer; %, percentage; SD, standard deviation; IQ, interquartile interval.
