# Supplementary material for: Built environment and physical activity in adolescents: Use of the kernel density estimation and the walkability index
Source: PLoS One. 2024 Mar 19;19(3):e0299628. doi: 10.1371/journal.pone.0299628 (PMC10950253; doi:10.1371/journal.pone.0299628)
Supplement: S2 Table — SD, standard deviation; IQ, interquartile range. (DOCX) [file pone.0299628.s004.docx]

**Supplementary Table 2. Descriptive measures of the Kernel density estimative in the radius of 400m, 800m, 1200m e 1600m.**

| **Variables** | **Average ± SD** | **Minimum Value** | | **Maximum Value** | | **Average (IQ)** |  |
| --- | --- | --- | --- | --- | --- | --- | --- |
| *Kernel Density: 400 meters* | 0.98 (±1.17) | 0.01 | | 6.25 | 0.86 (0.29 – 1.75) | | |
| *Kernel Density:*  800 meters | 2.08 (±2.92) | 0.00 | | 16.88 | 1.66 (0.77 – 3.50) | | |
| *Kernel Density:*  1200 meters | 3.79 (±5.21) | 0.00 | 27.07 | | 2.97 (1.56 – 6.23) | | |
| *Kernel Density:* 1600 meters | 6.07 (±7.72) | 0.10 | 36.22 | | 5.81 (2.79 – 11.95) | | |

SD, standard deviation; IQ, interquartile range.
