# Supplementary material for: Built environment and physical activity in adolescents: Use of the kernel density estimation and the walkability index
Source: PLoS One. 2024 Mar 19;19(3):e0299628. doi: 10.1371/journal.pone.0299628 (PMC10950253; doi:10.1371/journal.pone.0299628)
Supplement: S3 Table — *statistical significance; †, reference category; SD, standard deviation; LPA, light physical activity; MVPA, moderate to vigorous physical activity; SS, socioeconomic status. (DOCX) [file pone.0299628.s005.docx]

**Supplementary Table 3.** **Average values and interquartile interval of the continuous variables and values of the absolute and relative frequency of the categorical variables, for the total sample and separated by sex.**

| **CONTINUOUS VARIABLES** | | | | | | | |
| --- | --- | --- | --- | --- | --- | --- | --- |
|  | **Total 292**  **(100%)**  **Average (± SD)** | | | **Female 167 (57.2%)**  **Average (SD)** | | **Masculine 125 (42.8%)**  **Average (SD)** | **p-value** |
| *Age (years)* | 15.38 (**±** 0.56) | | | 15.38 (**±** 0.57) | | 15.38 (**±** 0.55) | 0.92 |
| *LPA (min)* | 157.4 (**±** 35.8) | | | 153.6 (**±** 35.5) | | 162.5 (**±** 35.6) | 0.04* |
| *MVPA (min)* | 64.7 (**±** 26.4) | | | 56.1 (**±** 20.5) | | 76.0 (**±** 29.0) | <0.01* |
| *Steps/day* | 8437 (± 2703) | | | 7668 (± 2385) | | 9464 (± 2770) | <0.01* |
| **CATEGORICAL VARIABLES** | | | | | | | |
|  | | **Total 292**  **(100 %)** | | | **Female**  **167 (57.2%)** | **Masculine**  **125 (42.8%)** | **p-value** |
|  | | |  |  | |  |  |
| ***SES***  Low† | | |  |  | |  | 0.02* |
|  |  |  | 60 (20.6%) | 27 (16.2%) | | 33 (26.4%) |  |
| Medium | | | 153 (52.4%) | 93 (55.7%) | | 60 (48.0%) |  |
| High | | | 66 (22.6%) | 43 (25.7%) | | 23 (18.4%) |  |
| Missing | | | 13 (4.4%) | 4 (2.4%) | | 9 (7.2%) |  |

*statistical significance; †, reference category; SD, standard deviation; LPA, light physical activity; MVPA, moderate to vigorous physical activity; SS, socioeconomic status.
